# Synergistic activity of ALK and mTOR inhibitors for the treatment of NPM-ALK positive lymphoma

**DOI:** 10.18632/oncotarget.12128

**Published:** 2016-09-20

**Authors:** Sara Redaelli, Monica Ceccon, Laura Antolini, Roberta Rigolio, Alessandra Pirola, Marco Peronaci, Carlo Gambacorti-Passerini, Luca Mologni

**Affiliations:** ^1^ University of Milano Bicocca, School of Medicine, 20900 Monza, Italy; ^2^ Center of Biostatistics for Clinical Epidemiology, University of Milano Bicocca, School of Medicine, 20900 Monza, Italy; ^3^ San Gerardo Hospital, Hematology-Clinical Research Unit, 20900 Monza, Italy

**Keywords:** ALK/ALCL, synergy, TKI, targeted therapy, resistance

## Abstract

ALK-positive Anaplastic Large Cell Lymphoma (ALCL) represents a subset of Non-Hodgkin Lymphoma whose treatment benefited from crizotinib development, a dual ALK/MET inhibitor. Crizotinib blocks ALK-triggered pathways such as PI3K/AKT/mTOR, indispensable for survival of ALK-driven tumors.

Despite the positive impact of targeted treatment in ALCL, resistant clones are often selected during therapy. Strategies to overcome resistance include the design of second generation drugs and the use of combined therapies that simultaneously target multiple nodes essential for cells survival. We investigated the effects of combined ALK/mTOR inhibition. We observed a specific synergistic effect of combining ALK inhibitors with an mTOR inhibitor (temsirolimus), in ALK+ lymphoma cells. The positive cooperation resulted in an increased inhibition of mTOR effectors, compared to single treatments, a block in G0/G1 phase and induction of apoptosis. The combination was able to prevent the selection of resistant clones, while long-term exposure to single agents led to the establishment of resistant cell lines, with either ALK inhibitor or temsirolimus. *In vivo*, mice injected with Karpas 299 cells and treated with low dose combination showed complete regression of tumors, while only partial inhibition was obtained in single agents-treated mice. Upon treatment stop the combination was able to significantly delay tumor relapses. Re-challenge of relapsed tumors at a higher dose led to full regression of xenografts in the combination group, but not in mice treated with lorlatinib alone. In conclusion, our data suggest that the combination of ALK and mTOR inhibitors could be a valuable therapeutic option for ALK+ ALCL patients.

## INTRODUCTION

Rational combined therapy has been widely investigated in recent years as a strategy to increase the efficacy of targeted therapy and to prevent/overcome resistance, with several examples of positive cooperation between two drugs [1–4].

ALK+ Anaplastic Large Cell Lymphomas (ALCL) represent a defined subset of Non-Hodgkin lymphomas (NHL) characterized by chromosomal rearrangements involving the Anaplastic Lymphoma Kinase (ALK) [5]. The most frequent rearrangement (85% of cases [6]) involves nucleophosmin (NPM1) as the 5′ fusion partner and the resulting NPM-ALK fusion protein is characterized by abnormal ALK activation. ALK has also been described as rearranged or mutated in other cancer types [5, 7] and, in all the cases, its deregulated kinase activity leads to the activation of several downstream pathways such as MEK/ERK, STATs and AKT/mTOR, which result in abnormal proliferation and block of apoptosis. As the deregulated activity of ALK is crucial for the survival of cancer cells, ALK selective targeting has been pursued similarly to what happened for BCR/ABL in chronic myeloid leukemia. Crizotinib is the first tyrosine kinase inhibitor (TKI) successfully tested in ALCL patients [8] and in 2011 it was approved for the treatment of ALK+ non-small cell lung cancer (NSCLC) [9–11]. Unfortunately, resistance to crizotinib frequently arises, mainly caused by point mutations or bypass mechanisms, thus causing tumor relapse [12–14]. This has spurred the development of second generation TKIs [15–19], including alectinib (CH5424802) and lorlatinib (PF-06463922). Alectinib was developed to be more selective than crizotinib and active against known crizotinib-resistant ALK mutations, and it has recently been approved by FDA as second-line treatment of advanced ALK+ NSCLC who failed crizotinib [20]. Lorlatinib is a highly potent dual ROS1/ALK inhibitor that was designed to have a reduced P-gp interaction to achieve drug exposure in the CNS. Lorlatinib shows significant activity against all crizotinib-resistant ALK mutants and it is currently in phase 1/2 clinical trial in ALK+ and ROS+ NSCLC [21].

Mammalian target of rapamycin (mTOR) signaling has been reported among the altered pathways in ALK+ ALCL. As part of the mTORC1 multi-protein complex, mTOR stimulates protein synthesis and cell cycle progression via its downstream effectors 4EBP1 and p70S6K [22]. Given the importance of mTOR pathway, not only in ALK-related cancer, targeted therapy aimed to block its dysregulated downstream signaling has been widely investigated [23]. Among the drugs under study, temsirolimus, a rapamycin analog, acts by allosterically inhibiting mTORC1 complex and is approved for the treatment of renal cell carcinoma [24].

Considering the relevance of ALK activity and mTOR signaling for the survival and proliferation of ALK+ tumor cells, we decided to investigate the effects of the simultaneous targeting of ALK and mTOR pathways in ALK+ ALCL, in order to increase the antitumor efficacy of the single agents and to prevent the selection of resistant clones. We describe here the synergistic effect of this rational combination both *in vitro* in ALK positive cell lines as well as in an *in vivo* model of the disease.

## RESULTS

### ALK inhibitors and temsirolimus synergistically impair the proliferation of ALK+ cell lines

**Figure 1:**
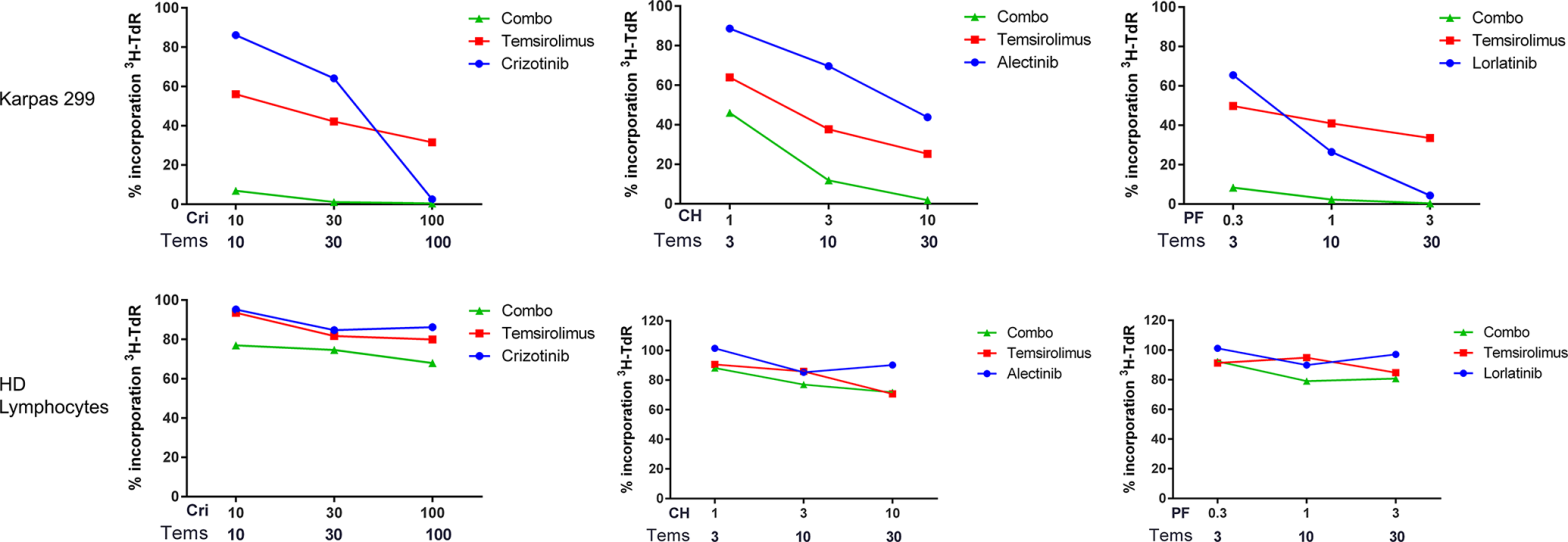
Evaluation of combined treatment effect on cellular proliferation Karpas 299 cells or normal lymphocytes were treated for 72 hours with selected nanomolar concentrations of ALK inhibitors and temsirolimus as single agents (ALK inhibitor alone, blue circles; Temsirolimus alone, red squares) or in combination (combo, green triangles). After 72 hours, tritiated thymidine incorporation was measured. Drug concentrations are indicated below the graphs: selected ratios, corresponding to those reported in Table 1, are shown. Each data point is normalized over the DMSO-treated control. Results are the average of at least 3 independent experiments performed in quadruplicate. Cri = Crizotinib, CH = Alectinib, PF = Lorlatinib, Tems = Temsirolimus.

The effect of the simultaneous inhibition of ALK and mTOR was assessed by combining two inhibitors across several ratios. Drug concentrations were chosen to be low enough in order to allow evaluation of synergistic/additive interactions. In each case a dose matrix was built, in which the IC50 values of the single agents were the central row and the central column, as suggested by Chou [25]. The treatment was carried out in three NPM-ALK positive ALCL cell lines for 72 hours and the proliferation rate was assessed. Three different ALK inhibitors (crizotinib, alectinib and lorlatinib) were used at low concentrations, either alone or in combination with temsirolimus as an mTOR inhibitor. Only for SUDH-L1 cells, a different temsirolimus concentration range was tested compared to other cells, mainly due to an intrinsic peculiar sensitivity to the single agent. In all the cases we observed a combined effect ranging from ‘synergism’ to ‘strong synergism’ as defined by Chou and Talalay [25] (Table 1, Figure 1, Supplementary Figure S1 and Supplementary Table S1). In order to exclude a possible unspecific, toxic effect of the combined treatment, we performed the same experiments in NPM-ALK negative cells derived from a healthy donor as well as in the NPM-ALK negative lymphoid tumor cell line U937 (Table 1, Supplementary Figure S1). In these settings, none of the combinations tested was synergic. These results indicate a possible beneficial effect of simultaneous targeting of ALK and mTOR, which is specific for NPM-ALK positive cells.

**Table 1:** Combination indexes from proliferation experiments

Crizotinib – Temsirolimus							
Cell lines		Ratio	Combination Index (CI)			Average CI	Synergism level
			EC50	EC75	EC90		
**NPM–ALK+**	Karpas 299	1:1	0.42	0.35	0.33	0.37	Synergism
	SUDH-L1	3:1	0.49	0.45	0.42	0.45	Synergism
	SUP-M2	1:1	0.59	0.33	0.29	0.40	Synergism
**NPM–ALK–**	U937	1:1	> 10	> 10	> 10	> 10	Antagonism
	HD Lymphocytes	1:1	> 10	> 10	> 10	> 10	Antagonism

Alectinib – Temsirolimus							
Cell lines		Ratio	Combination Index (CI)			Average CI	Synergism level
			EC50	EC75	EC90		
**NPM–ALK+**	Karpas 299	1:3	0.56	0.29	0.15	0.33	Synergism
	SUDH-L1	1:3	0.53	0.45	0.38	0.45	Synergism
	SUP-M2	1:10	0.65	0.18	0.06	0.3	Strong synergism
**NPM–ALK–**	U937	1:3	1.5	> 10	> 10	> 10	Antagonism
	HD Lymphocytes	1:3	1.2	2.7	7.2	3.7	Antagonism

Lorlatinib – Temsirolimus							
Cell lines		Ratio	Combination Index (CI)			Average CI	Synergism level
			EC50	EC75	EC90		
**NPM–ALK+**	Karpas 299	1:10	0.30	0.15	0.15	0.20	Strong synergism
	SUDH-L1	1:30	0.57	0.41	0.29	0.42	Synergism
	SUP-M2	1:3	0.30	0.12	0.06	0.16	Strong synergism
**NPM–ALK–**	U937	1:10	3.2	5.39	> 10	> 10	Antagonism
	HD Lymphocytes	1:10	0.73	3.68	> 10	5.5	Antagonism

Synergism levels are calculated according to Chou [25]. Results are the average of at least 3 independent experiments. HD = healthy donor.

### Dual ALK/mTOR inhibition inhibits cell signaling pathways and cell cycling

**Figure 2:**
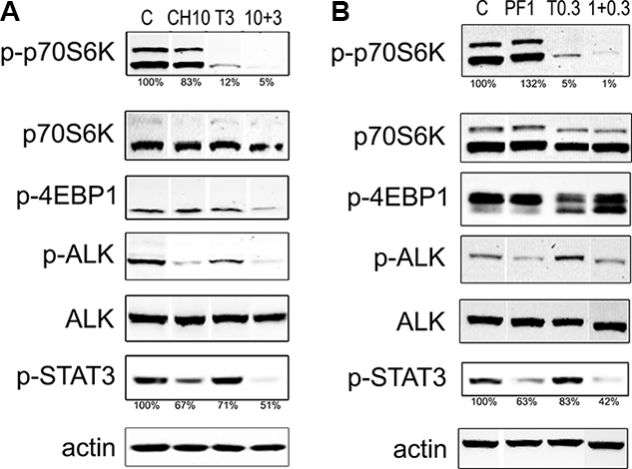
Immunoblot analysis of ALK/mTOR downstream pathways Two million Karpas 299 cells were treated for 4 hours with different concentrations of temsirolimus (T) and alectinib (CH) (**A**) or lorlatinib (PF) (**B**) as single agents or in combination. Whole cell lysates were loaded on a gel and probed with the indicated antibodies in western blot. Densitometry analysis of phospho-p70S6K and phospho-STAT3, were calculated relative to the control sample, normalized over the actin signal. The data are representative of two independent experiments. Lanes of interest derived from a single western blot image were juxtaposed.

To further elucidate the effect of the simultaneous inhibition of ALK and mTOR, we analyzed the status of the respective pathways by western blot. ALK inhibitors are known to act by abrogating the autophosphorylation of ALK, as well as the phosphorylation of downstream molecules. Temsirolimus binding to mTOR impairs the phosphorylation of downstream proteins such as p70S6K (also known as *RPS6KB1*) and 4EBP1 (*EIF4EBP1*). Western blot analysis of cells treated with single agents or with selected combinations is shown in Figure 2 and Supplementary Figure S2. As expected, ALK phosphorylation (pALK) was reduced upon treatment with ALK inhibitors, alectinib or lorlatinib. Phospho-p70S6K signal was markedly reduced by temsirolimus treatment, however the effect was enhanced when the two inhibitors were used in combination. Interestingly, STAT3 phosphorylation was strongly inhibited by the concomitant blockage of ALK and mTOR (Figure 2 and Supplementary Figure S2).

**Figure 3:**
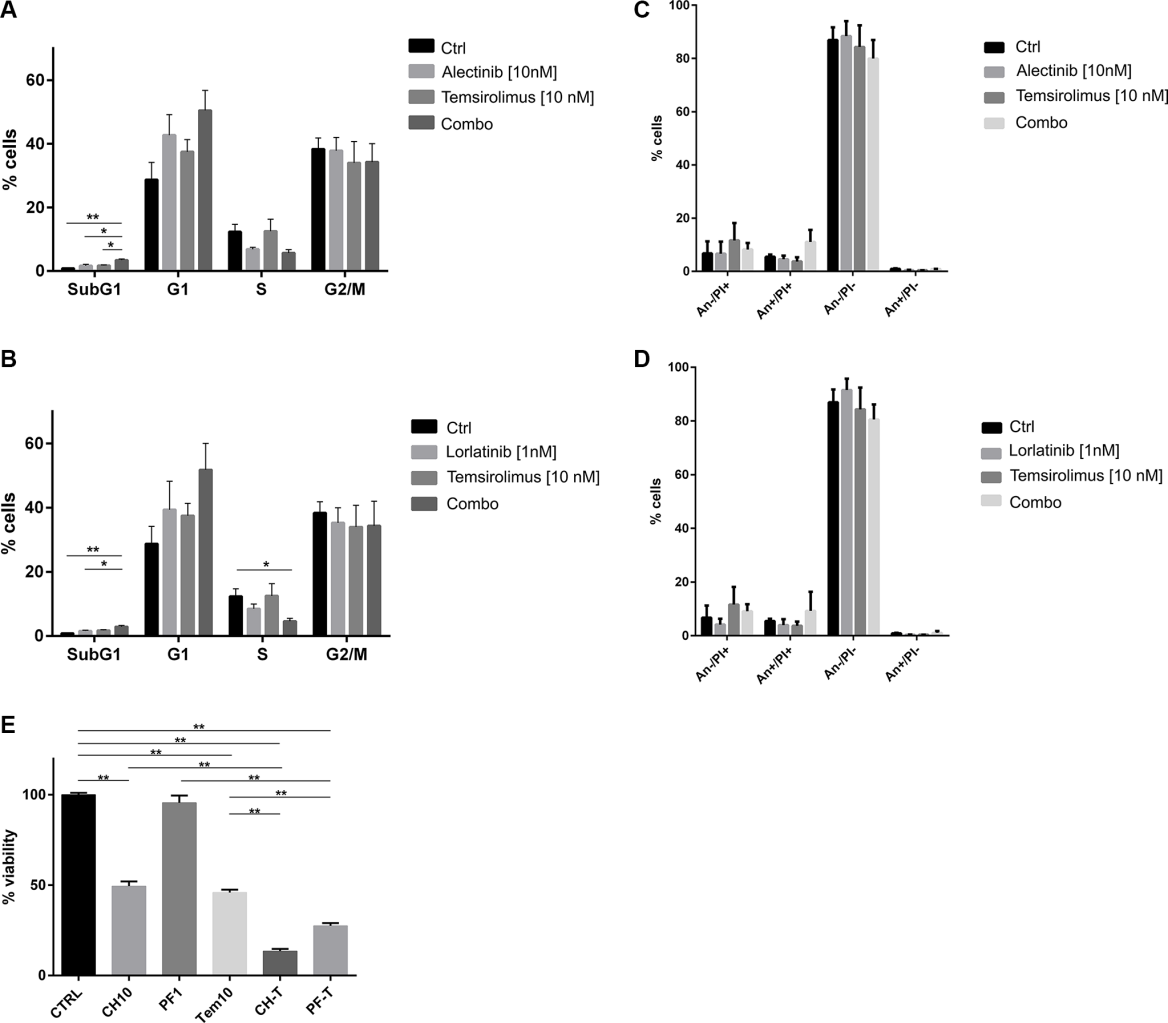
Effect of combined treatment on cell cycle Karpas 299 were treated up 96 hours with the indicated concentrations of temsirolimus, alectinib or lorlatinib either as single agents or in combination. Cell cycle analysis was evaluated after 72 hours with propidium iodide staining: (**A**) shows results for alectinib treated cells, (**B**) for lorlatinib treated cells. Results are the average of three independent experiments. (**C**, **D**) PI-Annexin V-FITC double staining on Karpas 299 samples performed in parallel to cell cycle analysis, after 72 hours of incubation with the drugs. Data presented refer to three independent experiments. (**E**) Karpas 299 viability was indirectly assessed by MTS assay after 96 hours of treatment. Percentage of viability normalized over the control sample is shown in the graph as average of 3 independent experiments ± SD For all the experiments *t-test* was performed to assess the statistical significance of the differences observed (*p-values < 0.05, **p-values < 0.01).

The effect of the combination on cell cycle distribution was assessed on Karpas 299 treated with selected concentration of ALK inhibitors, alectinib or lorlatinib, and temsirolimus as single agents or combined, up to 96 hours. At early time points (e.g. 48 h), a block in G1 was induced by the combinations, with no sign of cell death (data not shown). After 72 hours, cells treated with the two combinations showed a small but significant increase of the sub-G1 population, indicating cell death. At the same time, an increase in G1 phase compared to cells treated with single agents was observed, accompanied by a reduction of the S phase cell population (Figure 3A–3B, Supplementary Figure S3). Dual Annexin V-PI staining of treated cells confirmed the induction of apoptosis by combined treatments in ALK+ cells (Figure 3C–3D, An+/PI+ fraction). Finally, at 96 hours, cell culture viability was significantly reduced by dual ALK/mTOR inhibition, as measured by MTS assay (Figure 3E).

### Combined ALK/mTOR inhibition prevents the selection of drug-resistant cells

**Figure 4:**
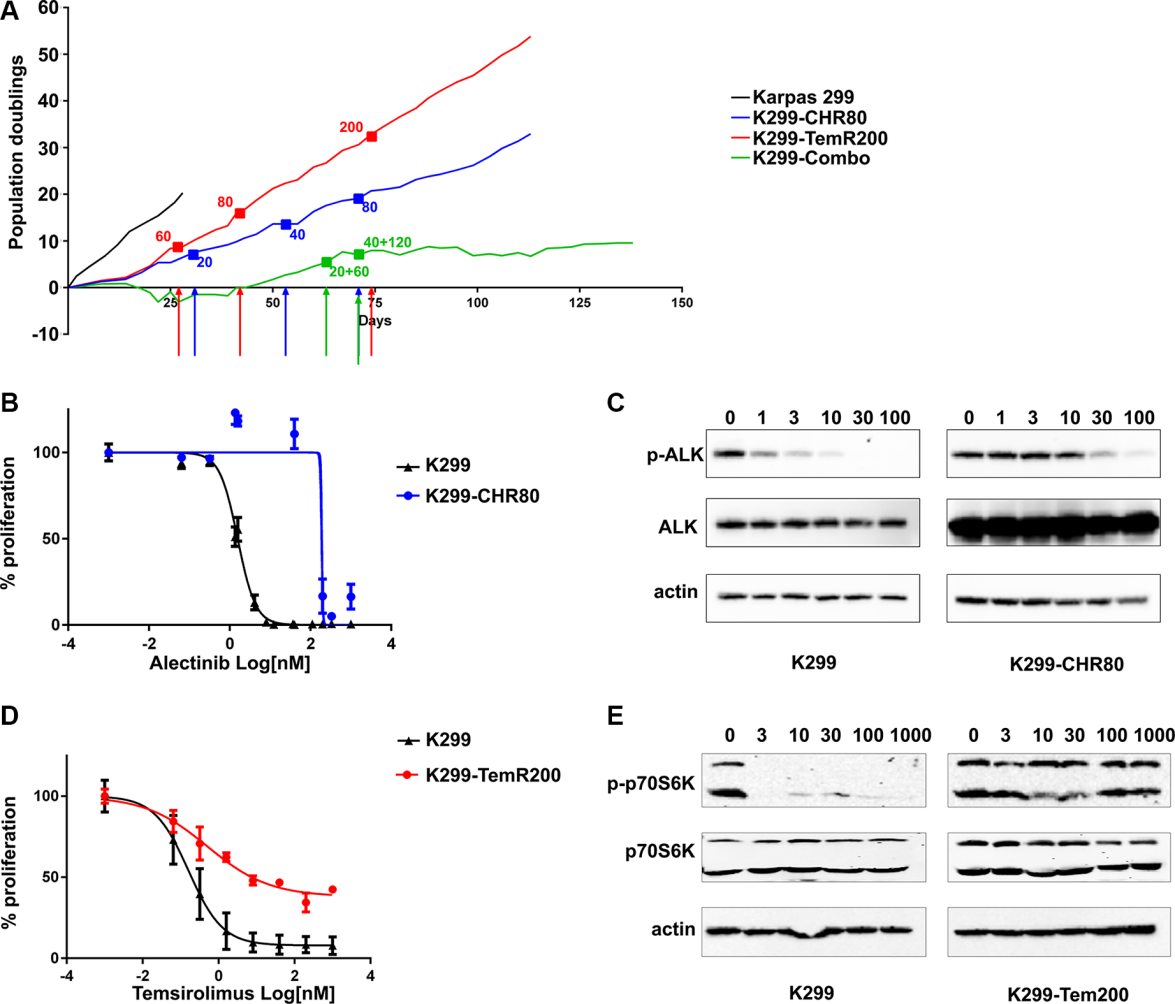
Selection and characterization of resistant cell lines (**A**) proliferation rate expressed as population doublings for Karpas 299 cells exposed to increasing concentrations of alectinib, temsirolimus or combination. Colored arrows indicate a dose escalation: red arrows correspond to temsirolimus, blue arrows to alectinib, green to the combination. The corresponding doses are indicated by a dot in the curves (**B**) dose-response curve of Karpas 299 cells resistant to alectinib (K299-CHR80) treated with alectinib. Parental Karpas 299 cells are used as control. Proliferation was assessed by tritiated thymidine incorporation assay after 72 hours of treatment. (**C**) western blot characterization of K299-CHR80 cell line treated with increasing concentrations of alectinib; comparison with parental Karpas 299. (**D**) dose-response curve of Karpas 299 cells resistant to temsirolimus (K299-TemR200) treated with temsirolimus. Parental Karpas 299 cells are used as control. Proliferation was assessed by tritiated thymidine incorporation assay after 72 hours of treatment (**E**) western blot characterization of K299-TemR200 treated with increasing concentrations of temsirolimus, compared to parental cells.

Acquisition of resistance represents one of the major issues in targeted therapy. To study if resistant clones could be selected as a consequence of continuous exposure to alectinib, we cultured Karpas 299 in the presence of increasing concentrations of the drug (Figure 4A) following the method described previously [12]. A resistant cell line was obtained (referred to as K299-CHR80) able to grow in the presence of 80 nM alectinib (50-fold the IC50 value of parental cells). The resistant cell line was characterized in order to determine the mechanism of resistance. Proliferation experiments confirmed resistance to alectinib: K299-CHR80 cells showed a 100-fold IC50 shift compared to the parental cell line (K299, 1.6 nM; K299-CHR80, 187 nM; Figure 4B). Western blot analysis confirmed persistence of NPM- ALK phosphorylation at high drug doses and showed a marked increase in NPM-ALK expression compared to the parental cell line, both at RNA and protein level (Figure 4C and Supplementary Figure S4A). No mutation in the ALK catalytic domain was detected by Sanger sequencing (data not shown), indicating that NPM-ALK overexpression is likely responsible for drug resistance [17]. In proliferation assay, combined treatment was effective also on K299-CHR80 (Supplementary Figure S4B). Similarly, we established a Karpas 299-derived cell line resistant to temsirolimus (K299-TemR200) (Figure 4A). Proliferation assay confirmed reduced sensitivity of K299-TemR200 to temsirolimus treatment compared to parental Karpas 299 cells (IC50: K299, 0.2 nM; K299-TemR200, 25 nM; Figure 4D). Interestingly, the dose-response curve of K299-TemR200 cells reached a plateau after 10 nM, that was maintained up to 1 μM temsirolimus, with a proliferation rate approximately 40% of the control. In line with proliferation data, western blot analysis performed on K299-TemR200 lysates clearly showed that phosphorylation of the mTOR effector p70S6K is not affected by temsirolimus concentrations up to 1 μM (Figure 4E). No mutation in FKB12 or mTOR was detected by Sanger sequencing (data not shown). At the same time, we examined whether the simultaneous exposure to alectinib and temsirolimus could prevent the selection of a resistant cell line. Indeed, although cells exposed to low-dose combination (10 nM alectinib plus 30 nM temsirolimus) did eventually grow out after 50 days, upon increased combined treatment with 40 nM alectinib and 120 nM temsirolimus (approximately half of single-selection doses) they were not able to expand (Figure 4A) and they showed a reduced viability compared to single agent treated cells (Supplementary Figure S4C). While control cells and cells under temsirolimus or alectinib selection achieved 10 population doublings in 13, 30 and 40 days, respectively, cells under dual selection did not reach this threshold up to 140 days.

**Figure 5:**
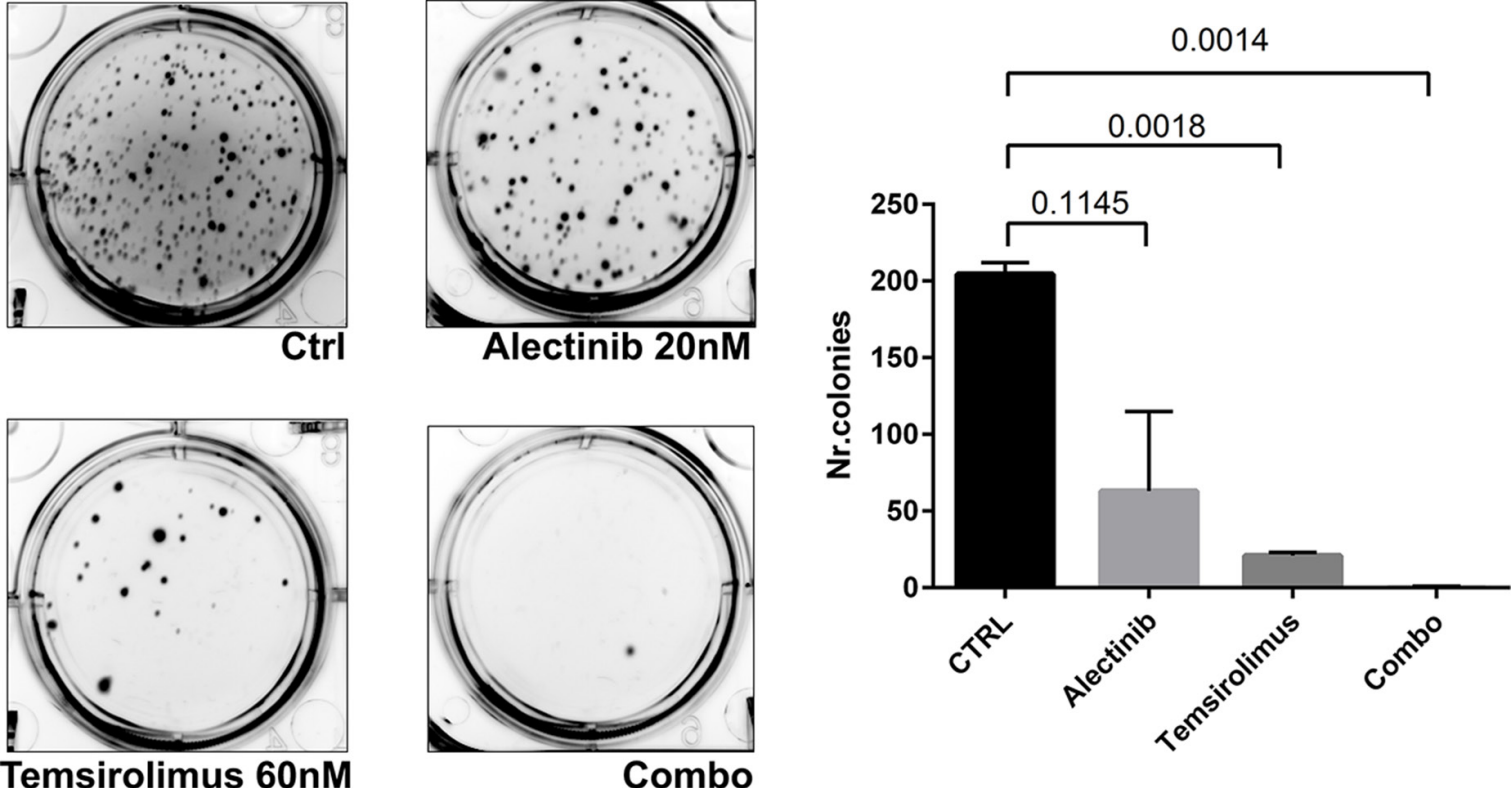
Effect of combined treatment on soft agar assay To explore the effects of long-term exposure to single or combined drugs on anchorage-independent growth, we performed a soft-agar colony assay on Karpas 299. Five thousands cells were seeded in 6-well plates, embedded in 0.3% low-melting agarose with or without different inhibitors concentrations as indicated on a 0.5% bottom agar layer, as previously described. Three weeks after seeding, colony formation was virtually suppressed by the simultaneous treatment with alectinib and temsirolimus.

To explore the effects of long-term exposure to single or combined drugs on anchorage-independent growth, we performed a soft-agar colony assay on Karpas 299. Three weeks after seeding, colony formation was virtually suppressed by the simultaneous treatment with alectinib and temsirolimus (Figure 5). Taken together, these results suggest that a combined treatment with ALK and mTOR inhibitors may prevent or greatly delay the development of resistance.

### Combined treatment induces robust tumor regression and delays relapses *in vivo*

**Figure 6:**
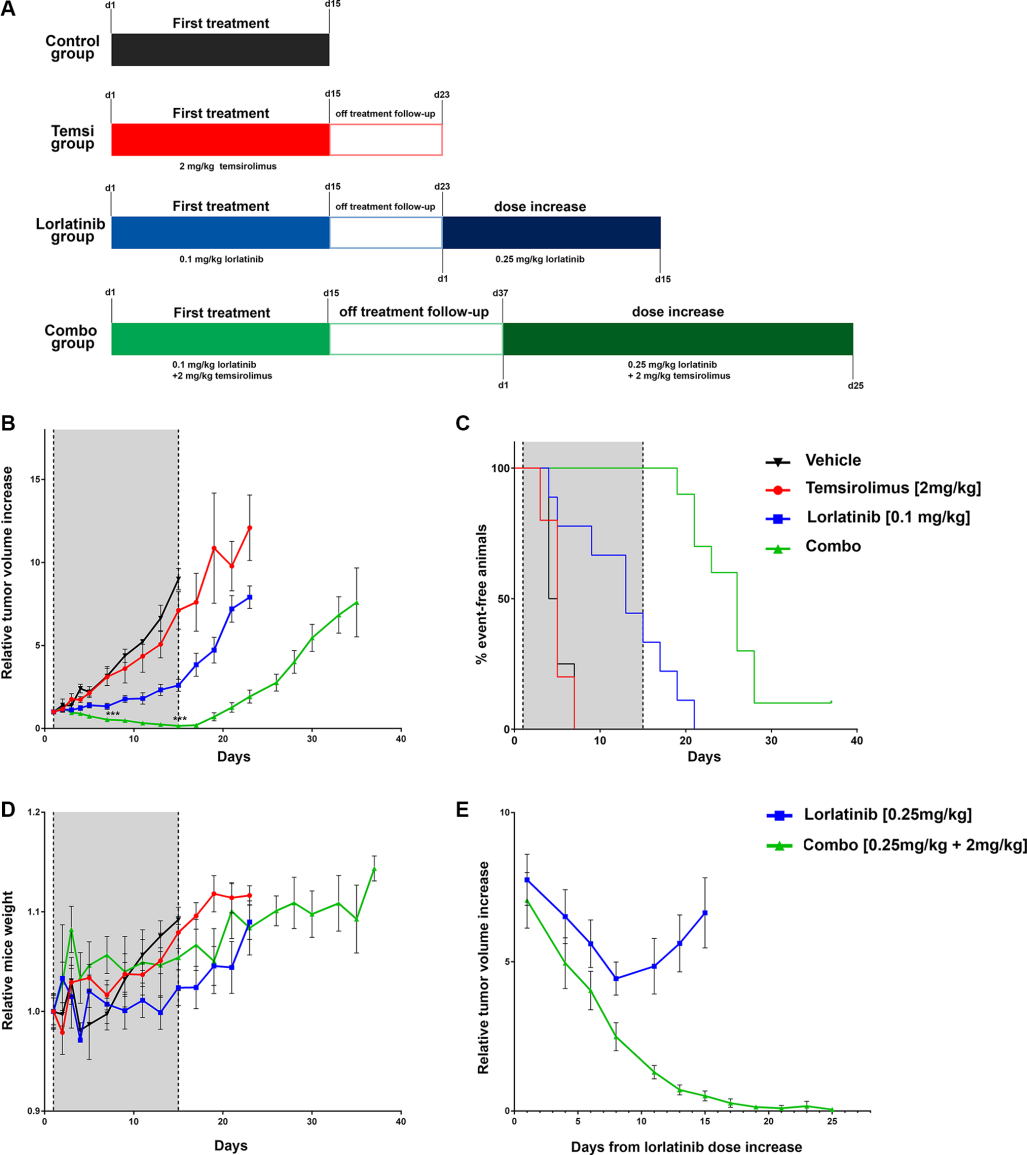
*In vivo* evaluation of the effect of combined treatment (**A**) summary of treatment schedule. For each group the doses and time of treatment are indicated. For lorlatinib and treatment combination group the two time scales (from treatment start and from dose increase) are reported. (**B**) relative tumor volumes in mice injected with Karpas 299 and treated with single agents, combination or vehicle only. For the graphical representation the tumor volume of each mouse was normalized over its volume at day 1. Shaded area indicates treatment period. Mean ± SEM is plotted. Mice receiving combination treatment showed a statistically significant reduction in the normalized tumor volumes compared to lorlatinib alone, both at day 7 (median = 0.53 vs 1.19 ***p-value = 0.003) and at day 15 (median = 0.15 vs 2.38,***p-value = 0.003. (**C**) Event-free survival analysis. The tumor growth for each mouse was monitored and normalized to its size at day 1. A two fold tumor increase was considered as an event. (**D**) Relative mice weight measurements. For the graphical representation the weight of each mouse was normalized over its weight at day 1. Mean ± SEM is plotted (**E**) relative tumor volumes during treatment increase. Lorlatinib was administered twice a day at 0.25 mg/kg either alone (8 mice) or in combination (8 mice) with temsirolimus [2 mg/kg]. The curves refer to the average normalized tumor volume.

To further validate our *in vitro* findings, Karpas 299 xenografts were grown subcutaneously in SCID mice and treated with lorlatinib, temsirolimus or a combination of the two drugs. Treatment started as tumors reached an average volume of 200 mm^3^ and was carried out for 15 days (Figure 6A). During the treatment period, little effect on tumor size was observed for mice treated with temsirolimus alone: as expected, the tumor growth curve of this treatment group did not significantly differ from the control group (temsirolimus vs control, day 7 median = 614 mm^3^ vs 583 mm^3^, *p-value* = 0.91; day 15, median = 1380 mm^3^ vs 1642 mm^3^, *p-value* = 0.61). Lorlatinib alone was able to control the increase of tumor masses but did not cause tumor regression (lorlatinib vs control: day 7 median = 221 mm^3^ vs 583 mm^3^, *p-value* = 0.02; day 15, median = 488 mm^3^ vs 1642 mm^3^, *p-value* = 0.003). In contrast, mice receiving the treatment combination showed a highly significant reduction in tumor masses compared to lorlatinib alone treatment already after 7 days of treatment (combination vs lorlatinib: median = 95 mm^3^ vs 221 mm^3^, *p-value* = 0.001) and reached nearly complete regression of tumors at day 15 (median = 25 mm^3^ vs 488 mm^3^, *p* = 0.00002) (Figure 6B and Supplementary Figure S5A). Analysis of individual responses indicated that all tumors treated with the combination regressed, while all but one lorlatinib-treated mice showed disease progression (Supplementary Figure S5B).

Upon treatment interruption, tumor masses of mice treated with lorlatinib alone progressively increased: after 8 days of follow-up (day 23 from treatment start; Figure 6A) the average relative tumor-size for the lorlatinib group was more than 7-fold the initial (treatment start) average tumor size (ratio to initial size: min = 3.7, max = 11.8 median = 7.9; Figure 6B and Supplementary Figure S5A), while combination-treated mice had a median size of 1.7x relative to initial size (min = 0.05, max = 3.74) that was significantly smaller than lorlatinib group (*p-value* = 0.00001). In the combination arm a comparable size to the one reached by the lorlatinib group was observed only at day 37 (median 7.7 fold increase).

We also considered for each mouse the time span from treatment start to the doubling of the initial tumor volume (Figure 6C). Mice treated with the combination remained event-free during treatment. They eventually doubled the initial size during the follow up, but tumors increase was significantly delayed compared to lorlatinib group (median time to doubling = 26 days for combination, 13 days for lorlatinib, *p-value* = 0.00005).

No significant variation in body weight within the treatment groups was observed at day 15 (treatment stop). Only lorlatinib as single agent showed a modest decrease in relative weight when compared to the control group (median = 1.01 vs 1.09, *p* = 0.02) (Figure 6D and Supplementary Figure S5C).

Subsequently, we wanted to assess whether the mice could benefit from an increased dose of lorlatinib (0.25 mg/kg) alone or in combination with the standard dose of temsirolimus. Eight mice from the lorlatinib group (median normalized tumor size 7.9 fold the initial volume, day 23) were shifted to increased lorlatinib dosage and 8 mice from the combination group (median tumor size 7.7, day 37) received the new combination with increased lorlatinib (Figure 6A, 6E and Supplementary Figure S5D). At day 1 after increase no significant difference in tumor masses was detectable (median lorlatinib 1492.16 mm^3^, combination = 1195.2 mm^3^, *p-value* = 0.44). Interestingly, while mice receiving lorlatinib alone initially responded to the new regimen, they all relapsed within 10 days: indeed, after 15 days of treatment increase, the median tumor size was 895 mm^3^ (median normalized tumor size, 4.8 fold the initial volume), while mice receiving the combination showed a marked decrease of the tumor masses (median tumor size 111 mm^3^, median normalized tumor size 0.6 fold the initial volume) that were significantly smaller than those of lorlatinib (*p-value* = 0.0002).

At day 25 since lorlatinib increase in 7 out of 8 mice of the combination group we observed a complete regression of the tumors that was sustained for at least 3 consecutive measurements (min = 0 mm^3^, max = 236 mm^3^, median 0 mm^3^) (Figure 6E and Supplementary Figure S5D).

## DISCUSSION

ALK positive ALCL therapy has benefited from crizotinib development, the first ALK inhibitor to be successfully used in patients. Subsequent development of next-generation ALK inhibitors such as alectinib and lorlatinib was aimed to improve the efficacy and overcome the resistance observed in a significant fraction of patients treated with crizotinib. The combination of two drugs has been widely explored in the last years as an effective strategy to overcome the onset of resistant clones in several tumors. The combination of crizotinib with a selective mTOR inhibitor has been recently described in ALK-mutated neuroblastoma [4] and NSCLC [26].

In the present work, we investigated the possibility to increase the efficacy of ALK inhibitors in ALCL, by simultaneously blocking the downstream mTOR pathway. The experiments were carried out using crizotinib as well as two second generation ALK inhibitors, alectinib and lorlatinib. It should be noted that all experiments were performed using low inhibitors doses. Combined drugs cooperatively decreased the proliferation rate of all the ALK positive ALCL cell lines tested, with an effect defined as synergistic to strongly synergistic, depending on the ALK inhibitor and the cell line. Interestingly, the observed positive cooperation was not detected in normal lymphocytes or in the NPM-ALK negative U937 cell line, thus excluding an unspecific toxic effect. The observed difference in the synergism strength could be due to the different genetic background of the cell lines [27]. The lack of any effect of the combinations in NPM-ALK-negative cells is explained by the fact that ALK inhibitors are almost completely inactive in this context, thus they cannot contribute to proliferation inhibition. To evaluate the effect of the combined treatment in the signaling cascade, we analyzed the phosphorylation status of some known targets of mTOR pathway. In all cases, p-p70S6K and p-STAT3 signal reduction observed in western blot was greater for the combination than for the single treatments, in line with the combination index values obtained (Table 1). While ALK inhibitor alone already decreased p-STAT3, it was insufficient to impact on p-p70S6K at the concentrations used here. Likely, the difference is due to the fact that STAT3 is a direct and p70S6K an indirect NPM-ALK target. Similarly temsirolimus directly blocked p-p70S6K but did not affect p-STAT3. However, both targets were significantly affected by drug combinations, indicating that complete shutdown of the pathway is needed to achieve superior anti-proliferative response. The behavior of p-4EBP1 was somewhat inconsistent through the various experiments, suggesting that either drug concentrations were too low, or p-4EBP1 is also controlled by alternative pathways in these cells. We could not use higher concentrations because we would have lost the synergistic effects. However, it has been reported that 4EBP1 phosphorylation at Thr37/46 is fairly insensitive to rapamycin, yielding inconsistent results [28].

At the cell cycle level, mTOR inhibition has been described to block cells in G1 phase [29]. Accordingly, our analysis of cell cycle distribution showed a block in G1 phase and a corresponding reduction of the S-phase population in cells treated with temsirolimus compared to the untreated cells. Such an effect was enhanced in cells treated with both an ALK inhibitor and temsirolimus and analyzed at different time points, up to 96 hours. The analysis of cell cycle distribution and apoptosis at 72 hours showed an increased induction of cell death in cells receiving the double treatment. Additional experiments performed with higher doses of the drugs led to a marked increase of dead cells already in the single-treated populations (not shown). This strong effect for the single agents, however, prevents the possibility of observing synergism at high concentrations. Taking these data and the *in vivo* dose escalation results altogether, it is likely that the combination of low doses of the drugs has a mixed cytostatic-cytotoxic effect, while higher doses have a more prominent cytotoxic effect.

Combination studies have been suggested as a possible way to reduce the selection of resistant clones that may arise as the result of suboptimal efficacy of single agents. The rationale behind this concept is that a cancer cell is less likely to simultaneously acquire two resistance ‘hits’. Thus, we investigated the behavior of our combined treatment compared to single agents in the selection of resistant cell lines. Cells exposed to either alectinib or temsirolimus alone acquired resistance over time as testified by the ability to grow in the presence of high concentrations of the drugs. Conversely, cells exposed to the combination were unable to proliferate thus indicating that the use of two drugs prevents the development of resistant clones. To further validate this finding, a soft agar colony assay shows that Karpas 299 cells treated with the drugs combination form a number of colony significantly reduced as compared to untreated cells.

To evaluate the efficacy of the combination in an *in vivo* model of ALCL, we treated for 2 weeks SCID mice subcutaneously injected with Karpas 299 with the two drugs as single agents or in combination. At the selected doses, lorlatinib was only able to control the tumor growth, while the combination treatment was significantly more effective in reducing the tumors size already after 7 days of treatment and resulted in an almost complete tumor regression at day 15. Upon treatment stop, all the tumors eventually increased their volume, but, interestingly, in the combination-treated mice we observed a significant delay in tumor relapse compared to lorlatinib alone. Importantly, no toxic effect of the combined treatment, measured as weight loss, was observed during the treatment. The only significant difference observed in lorlatinib alone group compared to the control group is likely due to the increased weight of control mice due to the large tumor masses (Supplementary Figure S5B).

We also investigated the effect of an increased dose of lorlatinib (0.25 mg/kg) in both groups. Interestingly, the combination resulted in a sustained complete regression of the relapsed tumors in 7/8 mice, while the single treatment was able to only partially reduce the tumor burden, that remained anyway measurable in 8/8 mice treated. Moreover, the effect of lorlatinib alone was short-lived, as all tumors restarted to grow under the increased dose treatment.

ALK is able to directly activate STAT3 pathway that indeed plays a pivotal role for the survival and proliferation of ALK driven tumors [5]. Moreover, ALK can activate mTOR (through PI3K-AKT and RAS-MAPK signaling) that is in turn able to promote indirect STAT3 activation [30]. The simultaneous inhibition of two potent upstream activators of STAT3, such as ALK and mTOR should result in a strong decrease of pSTAT3 signal, thus blocking the pro-survival signaling. Our experimental evidence support this hypothesis as shown in Supplementary Figure S6.

We describe here for the first time the *in vitro* and *in vivo* efficacy of a combined ALK-mTOR inhibition for the treatment of ALK+ ALCL. These findings provide a first indication that the combination of low-dose ALK and mTOR inhibitors may be beneficial for the treatment of this disease, by enhancing efficacy while reducing toxicity.

## MATERIALS AND METHODS

### Chemicals and reagents

Crizotinib, temsirolimus and lorlatinib (PF-06463922) were provided by Pfizer Inc., Alectinib (CH5424802) was purchased from Selleck Chemicals. All compounds were dissolved in DMSO (Sigma Chemical Co., St. Louis, MO) to obtain a 10 mmol/L stock solution, aliquoted and stored at −20°C for subsequent use. Tritiated thymidine was from Perkin Elmer (Waltham, MA).

### Cell lines and culture

ALCL NPM-ALK+ cell lines Karpas 299, SUDH-L1 and SUP-M2 were purchased from DSMZ, where they are routinely verified using genotypic and phenotypic testing to confirm their identity. Cells were cultured in RPMI 1640 (Euroclone) supplemented with 10% Fetal Bovine Serum (FBS), 2 mmol/L L-glutamine, 100 units/mL penicillin G, 80 μg/mL gentamicin, and 20 mmol/L HEPES, in a humidified atmosphere at 37°C and 5% CO_2_. As normal cells we used lymphocytes from healthy donors obtained culturing cells with 2.5 μg/ml Phytohemagglutinin-M (PHA-M) (Roche Diagnostics GmbH, Germany) and 200 UI/ml Interleukin-2 (IL- 2) (Aldesleukin, Novartis - Switzerland) for 3–4 days followed by 2–3 weeks incubation with IL-2 only.

### Proliferation assay and cell cycle analysis

Cells were seeded at a concentration of 10^4^ cells/well in 96-well round bottom cell culture plates with complete medium in the presence of increasing concentrations of inhibitors. Cell proliferation was measured at 72 hours using the tritiated thymidine incorporation assay as described previously [31]. The MTS (3-(4,5-dimethyl-2-yl)-5-(3-carboxymethoxyphenyl)-2-(4-sulfophenyl)-2H-tetrazolium, inner salt) assay was used to determine the number of viable cells, as an indirect method to assess viability, and was performed with the CellTiter 96^®^ AQ_ueous_ One Solution Cell Proliferation Assay (Promega) according to manufacturer's protocol. Cell cycle was analysed by flow cytometry and assessed with Propidium iodide (Sigma Chemical Co., St. Louis, MO), according to conventional techniques. PI-Annexin V-FITC staining (Bender Med System GmbH, Vienna, Austria) was performed accordingly to manufacturer protocol and analysed by flow cytometry on FACSCanto I (BD).

### Immunoblotting analysis

For immunoblotting analysis, 2 × 10^6^ cells were incubated for 4 hours at 37°C with selected inhibitors concentrations. Cells were lysed and equal amount of protein were loaded on SDS-PAGE for subsequent immunoblotting analysis. The following primary antibodies were used: phospho-p70 S6 Kinase (Thr389), p70 S6 Kinase (49D7), ALK (31F12), phospho-ALK (Tyr1604), phospho-4EBP1 (Thr37/46) (236B4), phospho- eIF4B (Ser422), phospho STAT3 (Tyr705), STAT3 all purchased from Cell Signaling Technology (CST, Danvers, MA). Loading control was performed using anti-actin antibody (Sigma Chemical Co., St. Louis, MO).

### Soft-agar colony assay

Five thousands cells were seeded in 6-well plates, embedded in 0.3% low-melting agarose (Sigma Chemical Co., St. Louis, MO) with or without different inhibitors concentrations on a 0.5% bottom agar layer, as previously described [32].

### *In vivo* experiment

Young adult (6 weeks old) female *scid* mice *C.B.17/IcrHanHsd-Prkdc* were purchased from Envigo Laboratories (San Pietro al Natisone, Udine, Italy) and kept under standard conditions following guidelines by the University of Milano-Bicocca ethical committee for animal welfare. The protocol was approved by the Italian Ministry of Health. Temsirolimus was dissolved in 100% ethanol (50 mg/ml) and stored at −20°C. The stock solution was diluted 1:250 in PBS containing 5% PEG400 and 5% Tween80 for intra-peritoneal (i.p.) administration. Lorlatinib was prepared fresh as a suspension in 0.5% carboxymethyl-cellulose + 0.1% Tween80. Ten million Karpas 299 cells were injected subcutaneously (s.c., 5×10^7^/ml) into the left flank of the mice. Once the tumors reached the average size of 200 mm^3^, mice were randomized (10 mice/group for combination and lorlatinib, 4/group for vehicle and 6/group Temsirolimus) and treated with temsirolimus (2 mg/kg every other day, i.p.), lorlatinib (0.1 mg/kg, administered *per os* twice a day), or both drugs. Control mice received vehicle alone. Tumor size was evaluated three times a week with a calliper, using the following formula: tumor volume (mm^3^) = (d^2^ × D/2), where D is the longest and d is the shortest diameter. Mice body weight was also evaluated three times a week for the whole treatment duration. After 14 days, treatments were suspended and mice observed until relative tumor volume was approximately 7-fold the initial size. During the second challenge, mice of the lorlatinib group received 0.25 mg/kg b.i.d. (*per os*), while the combination-treated mice received lorlatinib [0.25 mg/kg administered *per os* twice a day) plus temsirolimus (2 mg/kg every other day, i.p.).

### Statistical analyses

The combination effect for proliferation experiments was calculated using CalcuSyn software according to the method described by Chou and Talay [25], in which a combination index value (CI) is calculated for two drugs and allows the quantification of synergism: CI < 1, = 1 or > 1 indicate synergistic, additive or antagonistic interactions, respectively. Curve fitting, *p*-values and other statistical analyses were performed using GraphPad Prism 6 and R software.

Data on tumour and mice weights were analysed both as raw values and as ratio with respect to the initial values. Continuous data were described by the calculation of median values and ranges. Testing according to treatment groups was performed by unpaired nonparametric Wilcoxon test based on ranks. Survival time from the beginning of the study to the observation of the tumor size doubled with respect to the initial value was calculated and considered as censored to the time of the last measure when doubling was not observed. Kaplan Meier survival curves were used to describe survival data. Testing according to treatment groups was performed by the nonparametric log rank test. Two sided *p*-values were reported and considered significant when below the nominal 0.05 significance level.

## SUPPLEMENTARY MATERIALS TABLES FIGURES




