# Supplementary material for: Synergistic activity of ALK and mTOR inhibitors for the treatment of NPM-ALK positive lymphoma
Source: Oncotarget. 2016 Sep 20;7(45):72886–97. doi: 10.18632/oncotarget.12128 (PMC5341951; doi:10.18632/oncotarget.12128)
Supplement: Supplementary file 1 [file oncotarget-07-72886-s001.pdf]

Synergistic activity of ALK and mTOR inhibitors for the treatment of NPM-ALK positive lymphoma

Supplementary Materials

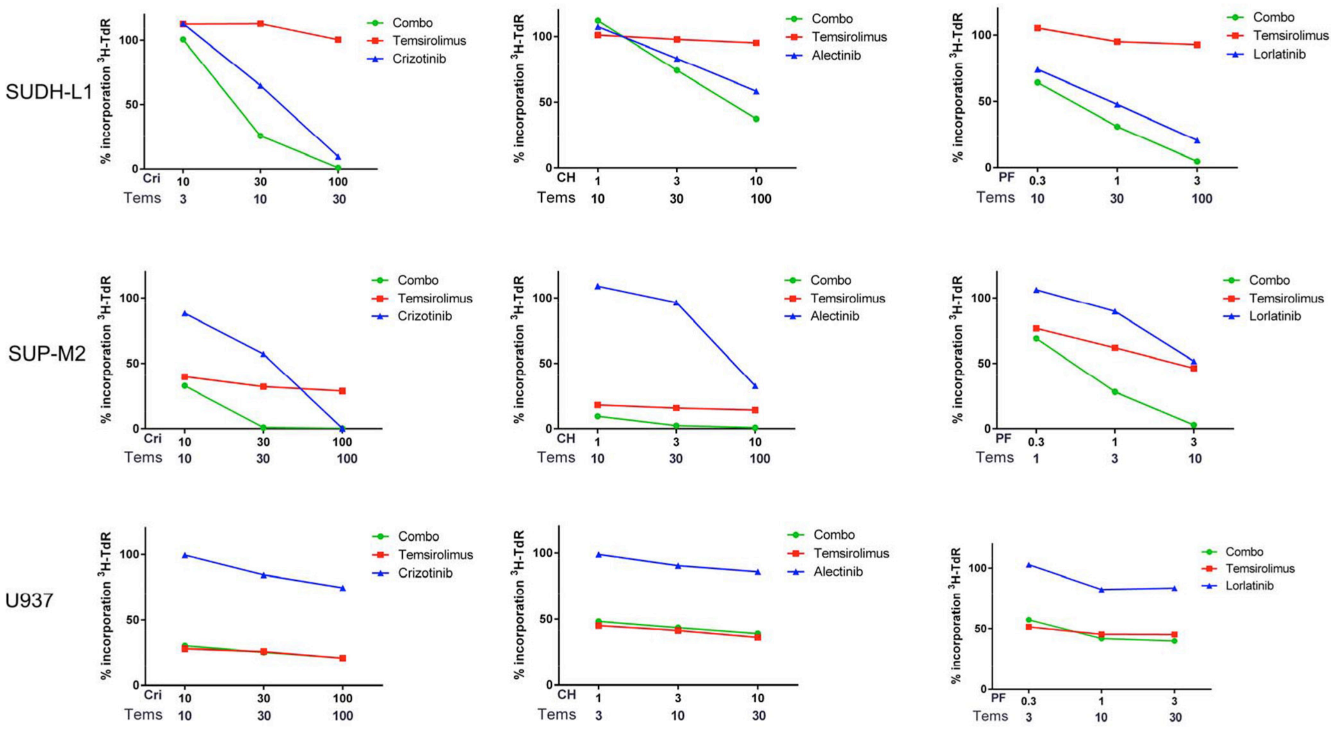

Supplementary Figure S1: Evaluation of combined treatment effect on cellular proliferation. ALK positive cell lines (SUDH-L1 and SUPM2) and ALK negative cell line (U937) were treated for 72 hours with selected nanomolar concentrations of ALK inhibitors and temsirolimus as single agents or in combination. After 72 hours tritiated thymidine (^{3}H-TdR) incorporation was measured. Selected ratios, corresponding to those reported in Table 1, are shown. Results are the average of at least 3 independent experiments performed in quadruplicate. Cri = Crizotinib, CH = Alectinib, PF = Lorlatinib, Tems = Temsirolimus.

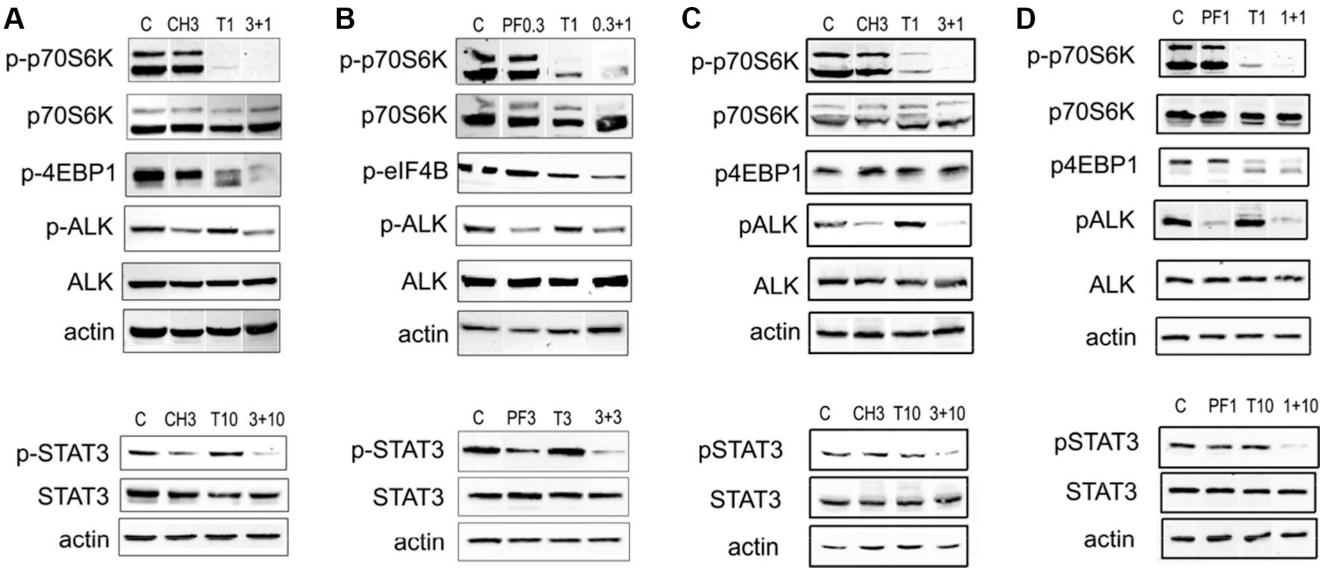

Supplementary Figure S2: Immunoblot analysis of ALK/mTOR downstream pathways. Two millions Karpas 299 (**A–B**) or SUP-M2 (**C–D**) cells were treated for 4 hours with the indicated nanomolar concentrations of temsirolimus (T) and alectinib (CH) (A, C) or lorlatinib (PF) (B, D) as single agents or in combination. Whole cell lysates were loaded on a gel and probed with the indicated antibodies in western blot. The data are representative of two independent experiments. Lane of interest derived from a single western blot image were juxtaposed.

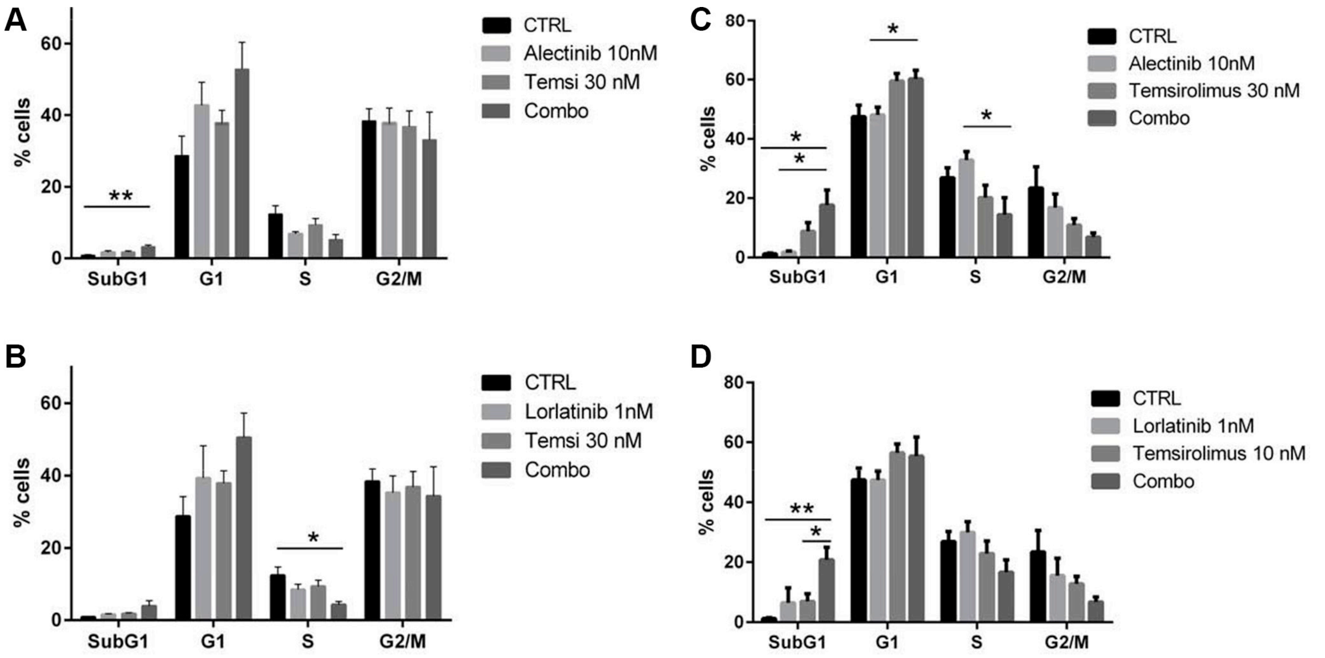

Supplementary Figure S3 : Effect of combined treatment on cell cycle. Karpas 299 (**A–B**) and SUPM2 (**C–D**) were treated for 72 hours with the indicated concentrations of temsirolimus, alectinib (A, C) or lorlatinib (B, D) either as single agents or in combination. Cell cycle analysis was evaluated with propidium iodide staining. Results are the average ± SEM of three independent experiments. For all the experiments *t*-test was performed to assess the statistical significance of the differences observed (**p*-values < 0.05, ***p*-values < 0.01).

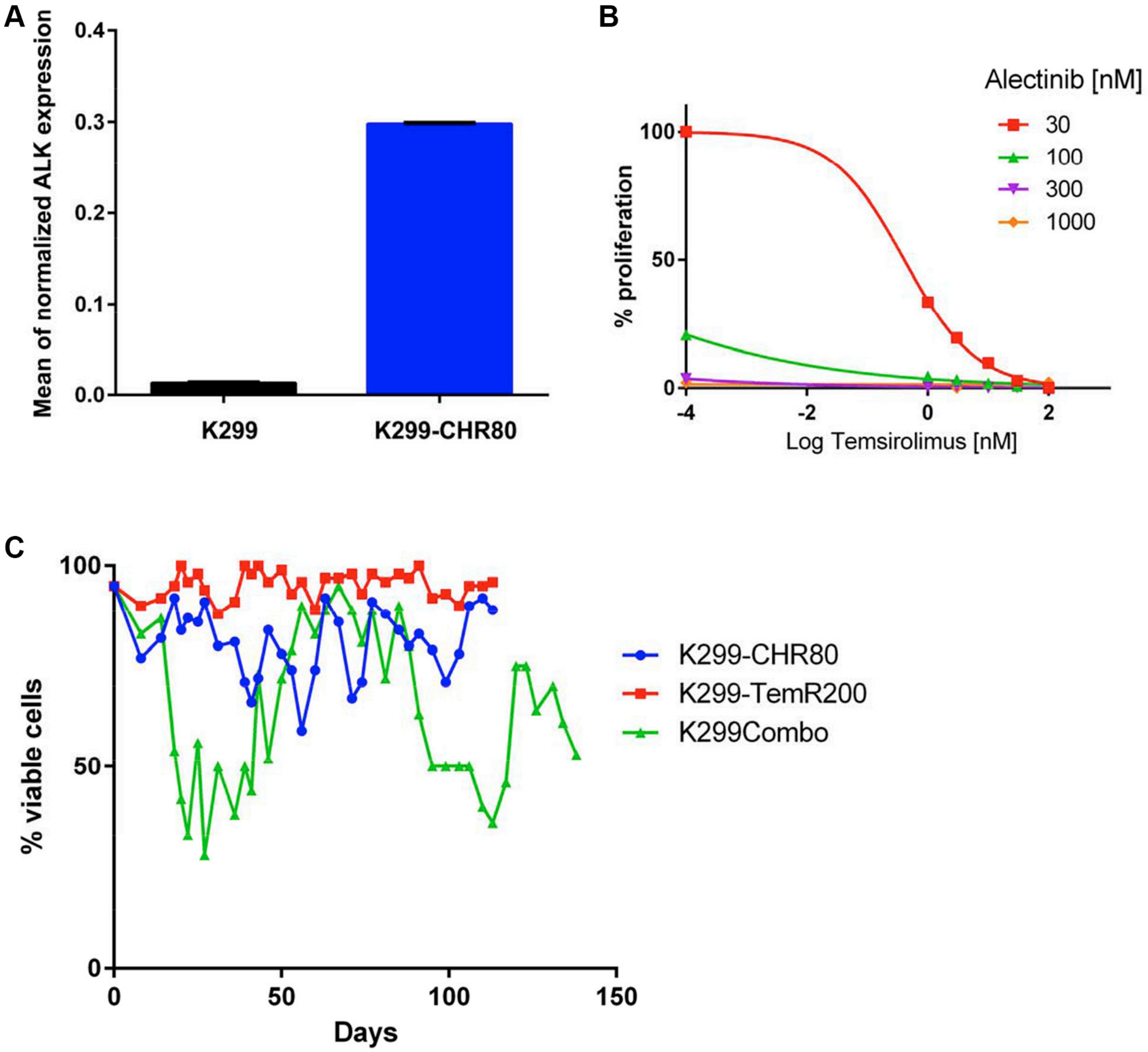

Supplementary Figure S4: Characterization of selected resistant cell lines. (**A**) NPM-ALK mRNA relative expression. Real-time qPCR performed on K299 parental and K299-CHR80, as described previously [1]. (**B**) Tritiated thymidine incorporation assay performed on K299-CHR80 after 72 hours of treatment with the indicated concentration of alectinib, temsirolimus as single agent or in combination. Cells are normally in culture in the presence of 30nM of alectinib, thus all the data are normalized over the proliferation value obtained in the presence of 30nM alectinib. (**C**) viability of the different cell lines during the selection. Trypan blue assay was performed regularly to evaluate the viability of the cell lines under selection with alectinib, temsirolimus and the combination of the two drugs. Results are expressed as percentage of viable cells over the total number of cells counted.

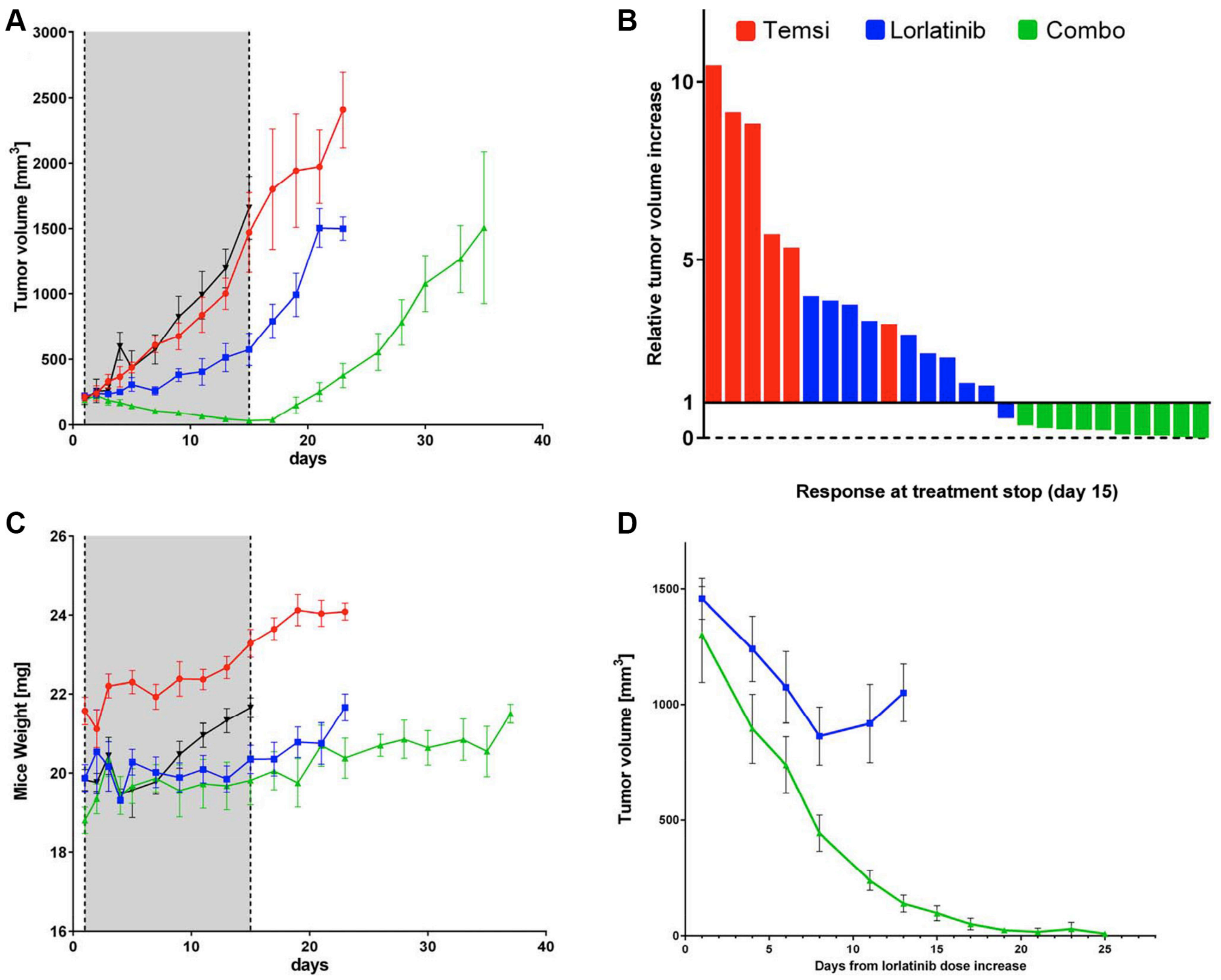

Supplementary Figure S5: *In vivo* evaluation of the effect of combined treatment. (**A**) tumor volumes (mm^3) in mice injected with Karpas 299 and treated with single agents (10 mice treated with lorlatinib, 6 mice with temsirolimus), combination (10 mice) or vehicle only (4 mice). Shaded area indicates treatment period. (**B**) Waterfall plot for the evaluation of the response in temsirolimus, lorlatinib and combination group after 15 days of treatment. For each mouse the tumor size is normalized on its size at the treatment start. (**C**) weight measurements expressed in mg. (**D**) tumor volumes (mm^3) for mice treated with the lorlatinib-increased (8 mice) or the combination-increased (8 mice) schedule. In all graphs, mean ± SEM is plotted.

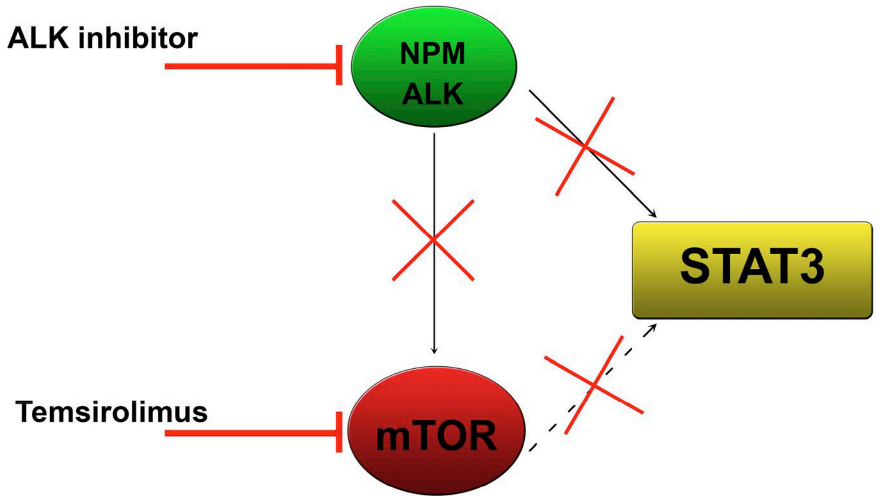

Supplementary Figure S6: Schematic representation of the ALK-mTOR- STAT3 interactions. The inhibitors effects on the pathway are highlighted with red crosses.

Supplementary Table S1: Full table of combination indexes obtained across all tested drug ratios in proliferation experiments. Synergism levels are calculated according to Chou [25]. Results are the average of at least 3 independent experiments. HD = healthy donor. See Supplementary_Table_S1

REFERENCES

1. Ceccon M, Mologni L, Giudici G, Piazza R, Pirola A, Fontana D, Gambacorti-Passerini C. Treatment Efficacy and Resistance Mechanisms Using the Second-Generation ALK Inhibitor AP26113 in Human NPM-ALK-Positive Anaplastic Large Cell Lymphoma. Molecular cancer research. 2015; 13:775–783.
