# Supplementary material for: Synergistic activity of ALK and mTOR inhibitors for the treatment of NPM-ALK positive lymphoma
Source: Oncotarget. 2016 Sep 20;7(45):72886–97. doi: 10.18632/oncotarget.12128 (PMC5341951; doi:10.18632/oncotarget.12128)
Supplement: Supplementary file 2 [file oncotarget-07-72886-s002.docx]

**Supplementary Table 1**: full table of combination indexes obtained across all tested drug ratios in proliferation experiments. Synergism levels are calculated according to Chou [25]. Results are the average of at least 3 independent experiments. HD = healthy donor.

| **Crizotinib -Temsirolimus** | | | | | |
| --- | --- | --- | --- | --- | --- |
|  | **Cell lines** | **Ratio** | **Combination index (CI)** | | |
|  |  |  | **ED50** | **ED75** | **ED90** |
| **NPM-ALK+** | **Karpas 299** | **1:1** | 0.42 | 0.35 | 0.33 |
|  |  | **1:3** | 0.67 | 0.46 | 0.35 |
|  |  | **3:1** | 0.47 | 0.40 | 0.38 |
|  |  | **10:1** | 0.60 | 0.56 | 0.54 |
|  | **SUDH-L1** | **1:1** | 0.45 | 0.50 | 0.55 |
|  |  | **3:1** | 0.49 | 0.45 | 0.42 |
|  |  | **10:1** | 0.50 | 0.47 | 0.44 |
|  |  | **30:1** | 0.53 | 0.49 | 0.46 |
|  | **SUP-M2** | **1:1** | 0.59 | 0.33 | 0.29 |
|  |  | **3:1** | 0.67 | 0.41 | 0.38 |
|  |  | **10:1** | 0.32 | 0.37 | 0.53 |
|  |  | **30:1** | 0.44 | 0.44 | 0.48 |
| **NPM-ALK-** | **U937** | **1:1** | >10 | >10 | >10 |
|  |  | **1:3** | >10 | >10 | >10 |
|  |  | **3:1** | 6.00 | >10 | >10 |
|  |  | **10:1** | >10 | >10 | >10 |
|  | **HD Lymphocytes** | **1:1** | >10 | >10 | >10 |
|  |  | **1:3** | 2.09 | 9.68 | >10 |
|  |  | **3:1** | >10 | >10 | >10 |

| **Alectinib -Temsirolimus** | | | | | |
| --- | --- | --- | --- | --- | --- |
|  | **Cell lines** | **Ratio** | **Combination index (CI)** | | |
|  |  |  | **ED50** | **ED75** | **ED90** |
| **NPM-ALK+** | **Karpas 299** | **1:1** | 0.51 | 0.26 | 0.13 |
|  |  | **1:3** | 0.56 | 0.29 | 0.15 |
|  |  | **1:10** | 0.47 | 0.29 | 0.19 |
|  |  | **1:30** | 0.36 | 0.24 | 0.23 |
|  |  | **3:1** | 0.56 | 0.28 | 0.14 |
|  |  | **1:1** | 0.54 | 0.46 | 0.39 |
|  |  | **1:3** | 0.53 | 0.45 | 0.38 |
|  |  | **1:10** | 0.42 | 0.45 | 0.49 |
|  |  | **3:1** | 0.61 | 0.50 | 0.41 |
|  | **SUP-M2** | **1:1** | 2.34 | 0.38 | 0.15 |
|  |  | **1:3** | 1.43 | 0.27 | 0.08 |
|  |  | **1:10** | 0.65 | 0.18 | 0.06 |
|  |  | **3:1** | 0.08 | 0.03 | 0.04 |
| **NPM-ALK-** | **U937** | **1:1** | 2.09 | >10 | >10 |
|  |  | **1:3** | 1.52 | >10 | >10 |
|  |  | **1:10** | 1.09 | >10 | >10 |
|  |  | **1:30** | 1.69 | >10 | >10 |
|  | **HD Lymphocytes** | **1:1** | 0.41 | 0.70 | 1.18 |
|  |  | **1:3** | 0.87 | 3.18 | >10 |
|  |  | **1:10** | 0.65 | >10 | >10 |
|  |  | **1:30** | 2.03 | 4.03 | >10 |

| **Lorlatinib -Temsirolimus** | | | | | |
| --- | --- | --- | --- | --- | --- |
|  | **Cell lines** | **Ratio** | **Combination index (CI)** | | |
|  |  |  | **ED50** | **ED75** | **ED90** |
| **NPM-ALK+** | **Karpas 299** | **1:1** | 0.47 | 0.35 | 0.30 |
|  |  | **1:3** | 0.25 | 0.19 | 0.19 |
|  |  | **1:10** | 0.30 | 0.15 | 0.15 |
|  |  | **1:30** | 0.62 | 0.16 | 0.14 |
|  | **SUDH-L1** | **1:1** | 0.78 | 0.60 | 0.47 |
|  |  | **1:3** | 0.68 | 0.51 | 0.38 |
|  |  | **1:10** | 0.63 | 0.44 | 0.31 |
|  |  | **1:30** | 0.57 | 0.41 | 0.29 |
|  | **SUP-M2** | **1:1** | 0.32 | 0.14 | 0.07 |
|  |  | **1:3** | 0.30 | 0.12 | 0.06 |
|  |  | **1:10** | 0.50 | 0.13 | 0.05 |
|  |  | **1:30** | 0.91 | 0.21 | 0.06 |
| **NPM-ALK-** | **U937** | **1:3** | 2.64 | >10 | >10 |
|  |  | **1:10** | 3.20 | 5.39 | >10 |
|  |  | **1:30** | 0.89 | >10 | >10 |
|  | **HD Lymphocytes** | **1:3** | 1.39 | 8.75 | >10 |
|  |  | **1:10** | 0.73 | 3.68 | >10 |
|  |  | **1:30** | 0.11 | >10 | >10 |
